# Real-Time Label-Free Surface Plasmon Resonance Biosensing with Gold Nanohole Arrays Fabricated by Nanoimprint Lithography

**DOI:** 10.3390/s131013960

**Published:** 2013-10-16

**Authors:** Josu Martinez-Perdiguero, Aritz Retolaza, Deitze Otaduy, Aritz Juarros, Santos Merino

**Affiliations:** 1 CIC microGUNE, Arrasate-Mondragón 20500, Spain; E-Mails: aretolaza@tekniker.es (A.R.); dotaduy@tekniker.es (D.O.); ajuarros@tekniker.es (A.J.); smerino@tekniker.es (S.M.); 2 Micro and Nanofabrication Unit, IK4-Tekniker, Eibar 20600, Spain

**Keywords:** label-free, biosensor, surface plasmon resonance, nanohole array, nanoimprint lithography

## Abstract

In this work we present a surface plasmon resonance sensor based on enhanced optical transmission through sub-wavelength nanohole arrays. This technique is extremely sensitive to changes in the refractive index of the surrounding medium which result in a modulation of the transmitted light. The periodic gold nanohole array sensors were fabricated by high-throughput thermal nanoimprint lithography. Square periodic arrays with sub-wavelength hole diameters were obtained and characterized. Using solutions with known refractive index, the array sensitivities were obtained. Finally, protein absorption was monitored in real-time demonstrating the label-free biosensing capabilities of the fabricated devices.

## 1. Introduction

Surface plasmon resonance (SPR) sensors are a common tool for real-time label-free biochemical interaction monitoring due to their ease of use and good performance. The Kretschmann configuration [1,2] is the most common operation method for commercial SPR systems requiring a somehow complex non-collinear optical setup. The use of enhanced transmission through metallic sub-wavelength nanohole arrays [2–4] to couple the incoming light to surface polaritons has proven to be a very good alternative because it makes use of a simple linear setup and boasts higher spatial resolution [5]. Both these characteristics allow for the miniaturization and micro-integration of the sensing device.

Ordered nanohole arrays are typically fabricated using focused ion beam (FIB), e.g., [3,6–8], or electron beam lithography (EBL) [9]. However, even though these techniques have very high resolution, the manufacture of arrays requires long processing times and, therefore, they are not suitable for mass-production due to their low throughput and the associated high cost. Alternative fabrication methods of ordered nanohole arrays have been reported in the literature such as nanosphere lithography [10] or phase-shifting photolithography [11].

**Figure 1.**
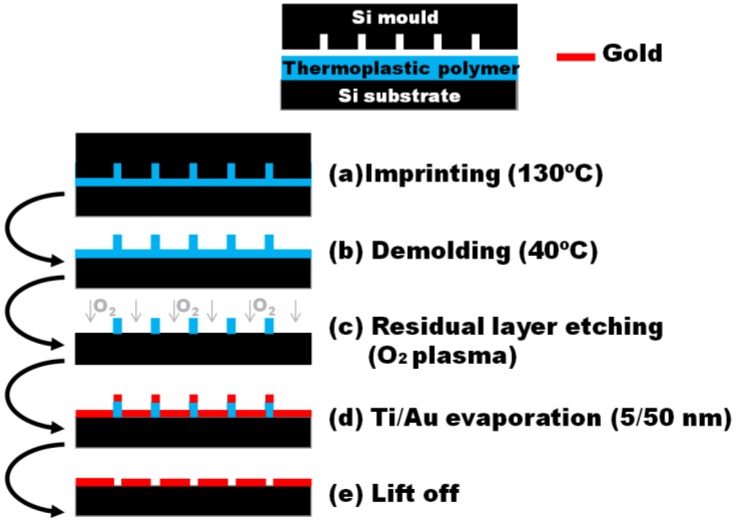
Schematic cross-section of the gold nanohole array fabrication process with thermal-NIL.

Nanoimprint lithography (NIL) is a mature technique which allows obtaining nanometer scale features on large size wafers [12]. In a standard thermal NIL process (see Figure 1), a thin layer of a thermoplastic polymer is spin coated on a substrate and pressured against a nanostructured mold. As the temperature is increased above the glass transition temperature of the polymer, it flows into the structures. Finally, after cooling down, a demolding process is carried out leaving the structures patterned on the resin. This pattern is then transferred to the substrate via an etching process. Due to its high throughput, low-cost, and high fidelity pattern transfer, it has great potential to be scaled up to real production. This enabling technology is considered a key candidate for the so-called next generation lithographies to be used in the low nanometric range, for example, in the semiconductor industry [13]. In [14], Chen *et al.* reported the fabrication of ordered nanohole arrays using UV-NIL by means of a trilayer imprint geometry and three steps of reactive ion etching (RIE). Im *et al.* used template-stripping to pattern the periodic nanohole arrays in Ag films with a silicon template made via nanoimprint lithography [15].

Here we report on the fabrication, characterization and demonstration of periodic gold nanohole square array sensors. High quality arrays were obtained on glass substrates using a thermal-NIL process on a single resist layer. The fabrication process was optimized and the arrays were fully characterized. The extraordinary optical transmission through the nanohole arrays was measured in an optical bench and the sensitivity was calculated using solutions of known refractive index. In addition, absorption of bovine serum albumina (BSA) protein onto the gold surface was monitored in real-time without the necessity of labels to demonstrate the biosensing capability of the fabricated device.

## 2. Experimental Section

### 2.1. Nanohole Array Fabrication Method

**Figure 2.**
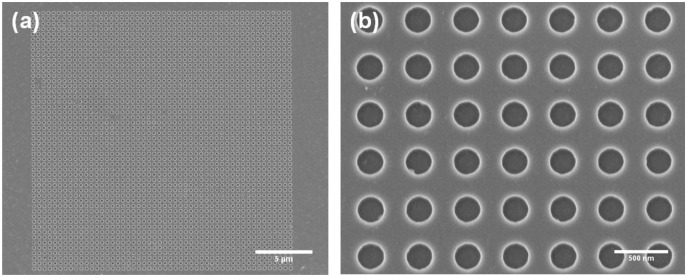
(**a**) and (**b**) SEM images of a master mold with nanohole square arrays (hole diameter 250 nm, periodicity 450 nm, depth 130 nm).

10.16 cm silicon masters were fabricated by EBL at the Institute of Optoelectronic Systems and Microtechnologies (CT-ISOM, Madrid, Spain). A 100 nm poly(methyl methacrylate) (PMMA) thin layer was spin coated on the 10.16 cm silicon wafer, afterwards the desired pattern was written by EBL (Cabl9000C, Crestec, Hachioji-shi, Japan). The structured PMMA was used as mask for a silicon dry etching process with SF6/C4F8 plasma (Plasmalab 80+, Oxford Instruments, Abingdon, United Kingdom). Finally, the silicon master was coated with tridecafluoro-(1,1,2,2)-tetrahydrooctyl trichlorosilane (ABCR GmbH, Karlsruhe, Germany) by vapor deposition for anti-sticking purposes during the imprinting process. Figure 2 shows scanning electron microscopy (SEM) images of a silicon master with a gold nanohole square array 450 nm periodicity, 250 nm hole diameter) with a total footprint of 23 × 23 μm^2^.

**Figure 3.**
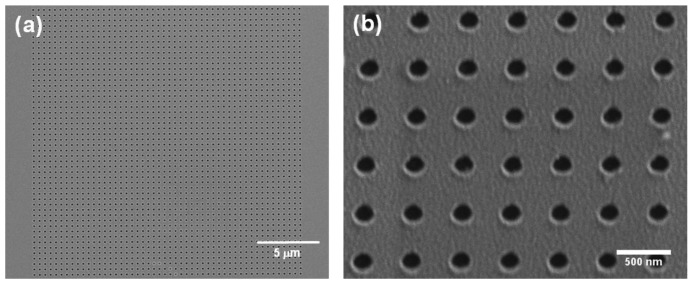
(**a**) and (**b**) SEM images of a gold nanohole array fabricated with NIL (hole diameter 185 nm, periodicity 450 nm, Ti/Au layer thickness 5/50 nm).

Gold nanohole arrays were obtained with the fabricated stamps by thermal-NIL, residual layer etching, Ti/Au deposition and lift-off processes (see Figure 1). The imprinting process was carried out on 1.1 mm thick 10.16 cm glass wafers (Vitrotec, Sant Andreu de la Barca, Spain) coated with a 100 nm layer of mr-I7010R thermoplastic polymer (Micro Resist Technology GmbH, Berlin, Germany). This layer was obtained by spin-coating at 3,000 rpm for 30 s in a Delta 20 W8 BM (BLE). The resist thickness was chosen to ensure an optimized filling of the cavities and minimize the residual layer thickness. Given that the polymer glass transition temperature was 50 °C, the NIL process was carried out applying a 20 kN pressure at 130 °C for 30′ in a HEX03 hot-embossing system (Jenoptik AG, Jena, Germany) as depicted in Figure 1a. After that, the demolding step was done at 40 °C (Figure 1b) and the residual layer was etched by O_2_ plasma (Plasmalab 80+, Figure 1c). Using this process, the polymer is homogeneously etched from above until the residual layer is completely removed and the pattern has been transferred to the substrate. A metallic Ti (5 nm adhesion layer)/Au (50 nm) layer was deposited by e-beam evaporation in an ATC Series Evaporation System (AJA International Inc., Boston, MA, USA) (Figure 1d). The metal layer thickness was chosen taking into account the metal layer to resist thickness ratio limitations of the lift-off process when a single resist layer is used. Finally, the resist lift-off process was carried out in an ultrasonic hot acetone bath to obtain the nanoholes array structures (Figure 1e). Gold chips were cleaned prior to every measurement by immersion during 180 s in a freshly prepared 3:1 H_2_SO_4_/H_2_O_2_ piranha solution, water rinsing and N_2_ drying. A SEM picture of an array fabricated with the stamp of Figure 2 is shown Figure 3.

Larger arrays (with footprints up to 600 × 600 μm^2^) with good homogeneity were also fabricated proving the imprinting over large areas. The array fabrication process with the optimized parameters was highly reproducible and the stamps were reused without array quality loss. It is worth mentioning a loss in the lateral dimensions, *i.e.*, diameter, of the imprinted nanocolumns during the residual layer etching RIE process probably due to non-perfectly anisotropic plasma. In the case of Figures 2 and 3, where the residual layer was 75 nm the hole diameter size reduction was quantified in c. 65 nm. This dimension loss is very likely avoidable using more anisotropic etching processes.

### 2.2. Optical Setup

**Figure 4.**
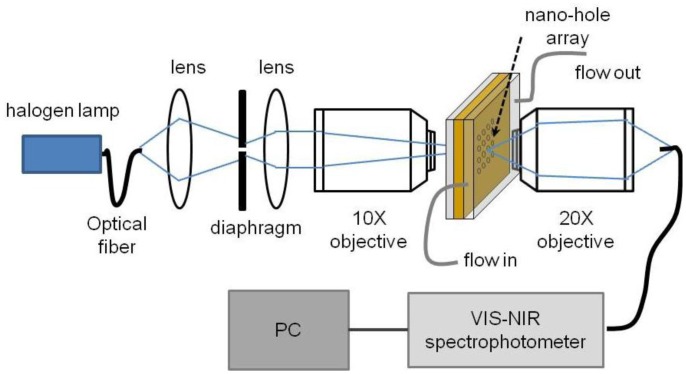
Scheme of the optical setup to measure the transmission spectra through the fabricated nanohole arrays.

The transmission spectra for the nanohole arrays were tested by using a 12 W broadband tugsten-halogen light source (HL-2000, Ocean Optics, Dunedin, FL, USA). Figure 4 shows the optical setup used for the experimental measurements. The light was spatially filtered using a lens, an iris diaphragm and a collimation lens, and was focalized to the gold chip with the nanohole array with a 10× objective. To avoid the influence of other features that may be in the chip, adjusting the size of the diaphragm, the beam cross-section at the focus was smaller than the array size. The light from the nanohole array was collected by a 20× objective and focused on a fiber optic coupled to a VIS-NIR spectrophotometer (HR-2000+, Ocean Optics). All spectra were obtained using normal incident and unpolarized light. The transmitted light intensity was digitally recorded, in counts, *vs.* wavelength in the range of 400 nm to 840 nm. The measurements were made in a controlled environment at 20 °C and a simple PDMS microfluidic cell was constructed to ease the measurement in liquid media.

## 3. Results and Discussion

### 3.1. Optical Properties of the Nanoholes Arrays

**Figure 5.**
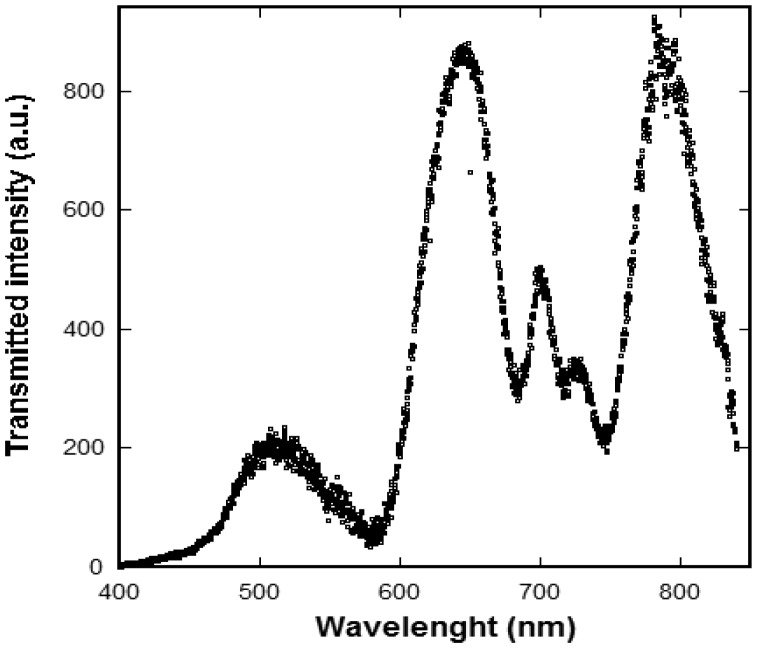
Modulated optical transmission through a gold nanohole array (that of Figure 3.) in air. The resonance peaks are the result of plasmon mediated transmission.

For the case of the square nanohole arrays fabricated and transmission of normally incident light the resonance wavelengths *λ_SP_* are given by [2–4]:

(1) $$\lambda_{SP}(i,j)=\frac{p}{\sqrt{i^{2}+j^{2}}}\frac{{ɛ}_{s}{ɛ}_{m}}{{ɛ}_{s}+{ɛ}_{m}}$$

where *i* and *j* are integers corresponding to the various resonance orders, *p* is the array periodicity and ε*_s_* and ε*_m_* are the metal and solution dielectric constants respectively. Figure 5 shows the modulated transmission spectrum of an array with 450 nm periodicity where several peak corresponding to different resonance orders are clearly observable.

**Figure 6.**
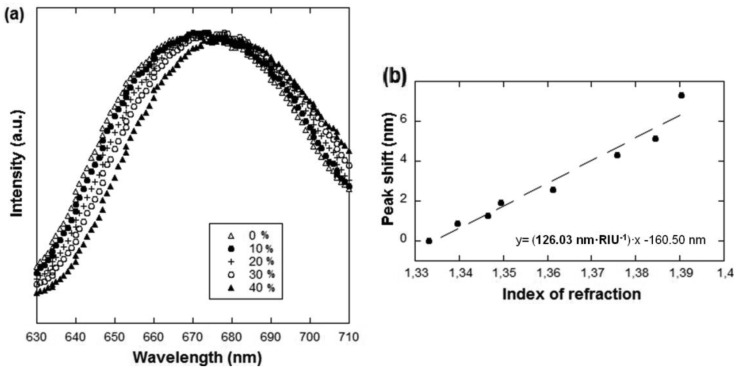
(**a**) Transmission spectra obtained for four sucrose solutions at different concentrations (see inset). A clear red shift with increasing concentration (or refractive index) can be observed; (**b**) Linear change of the peak wavelength position *vs.* solution refractive index. From this plot, a 126 nm/RIU sensitivity was obtained.

To characterize the light transmission through the nanohole arrays and to demonstrate chemical detection several solutions of known refractive indices were prepared. The refractive index of aqueous sucrose solutions with concentrations ranging from 0 up-to 40% were measured with a refractometer (DM40, Mettler Toledo, Columbus, OH, USA) and the light transmission spectra through the nanohole arrays in contact with these media recorded. Figure 6a shows the spectra obtained for four of these solutions using the array in Figure 3. As can be seen, as expected from the formula, there is a clear red shifting with increasing refractive index. Figure 6b shows the linear change of the peak wavelength position *vs.* solution refractive index. From the slope of this plot, a 126 nm per refractive index unit (nm/RIU) sensitivity was obtained, which is very close to the sensitivities of arrays with similar characteristics reported in the literature but fabricated with costlier techniques such as FIB or EBL (e.g., [4,5]). The sensitivity obtained is significantly smaller than the values obtained for non-nanostructured sensors using the typical Krestchmann configuration (of the order of 1,000 nm/RIU when using visible light). However, the number of biomolecules giving rise to the signal is much smaller in the case of the nanohole arrays due to the reduced footprint. The appeal of these sensors lies in this fact, together with the simpler collinear optical setup, because it offers great multiplexing and miniaturization possibilities.

### 3.2. Real-Time Label-Free Protein Absorption Monitoring

**Figure 7.**
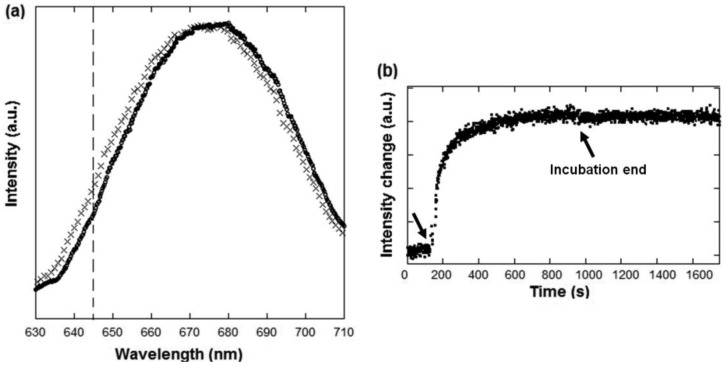
(**a**) Transmission spectra through a gold nanohole array (that of Figure 3) in contact with PBS buffer (crosses) and after 1 h incubation of a solution of 50 μg/mL BSA solution in PBS buffer (circles). The peak wavelengths *λ_SP_* are 673.1 nm and 675.9 nm respectively (**b**) Real-time label-free monitoring of the BSA absorption on the gold surface. The transmission intensity was monitored at a wavelength situated in the high-slope region of the peak (*λ* = 645 nm, see vertical line in (a)) for maximum sensitivity and linearity. The injection of BSA protein (arrow at *t* = 150 s) results in a change of the transmitted spectra which can be measured as an intensity change at a given wavelength. It can be observed that at after 800 s the surface was saturated with BSA and no further absorption took place. At t = 950 (see arrow) the buffer was switched back to PBS and no signal decrease was appreciable, proving the stability of the formed BSA layer.

In order to demonstrate the protein detection capabilities of the device, protein physisorption on the gold surface was monitored in real-time. To perform this task, BSA protein at a 50 μg/mL concentration in phosphate buffer solution (PBS) was incubated over the array and the spectrum was recorded continuously. Figure 7a shows the transmission spectra before (in pure PBS) and after a 10 min incubation of BSA. A 2.8 nm peak shift is measured which is of the order of what was expected for a protein layer in an array with the mentioned sensitivity (see, e.g., [6,7]). This shift remained after washing with PBS which proved that the developed sensor was not measuring a reversible bulk effect like the one obtained for the sucrose solutions (Figure 6)

In Figure 7b the transmitted intensity at a fixed wavelength (see vertical line in Figure 7a) is plotted *vs.* incubation time where the kinetics of the BSA absorption to the gold surface can be observed in a real-time label-free way. Initially the array was in contact with pure PBS buffer (baseline) and after 150 s the BSA in PBS buffer was injected and started absorbing on the surface. This process was followed monitoring the transmitted light intensity at 645 nm (where the intensity change was maximum). After c. 800 s, the signal did not further increase, proving that the surface was actually saturated with a BSA protein layer. After 950 s the buffer was again switched to pure PBS and no dissociation or bulk shift were measured, proving an stable physical absorption of the BSA protein on gold.

The measurement at a single wavelength shows the possibility of using more stable laser light illumination and an even simpler setup using a photodiode or CCD instead of a spectrometer [16] for biomolecular interaction monitoring. The linearity of the intensity signal change is guaranteed for shifts of a few nm expected for protein binding process if the wavelength is in the high-slope region of the resonance peaks (see Figure 7a) [17]. Parameters, such as the array periodicity, can be optimized for the use of, for instance, widely available He-Ne laser sources (633 nm) to enable further miniaturization and development of the sensing devices [16]. Moreover, it is worth mentioning that the sensing spot had a 530 μm^2^ footprint, which is, for instance, c. 60 times smaller than that of a typical DNA microarray spot (200 μm in diameter). This shows that high density multiplexing biosensing devices could be achieved.

## 4. Conclusions

We have showed the fabrication, characterization and demonstration of optical gold nanohole array sensors. The NIL fabrication process using a single resist layer has proved to be repetitive and the quality of the arrays obtained is similar to those manufactured using more common techniques in this field such as FIB or EBL which, however, lack the throughput of NIL. The light transmission properties of the arrays have been tested in a designed optical setup and chemical plasmonic sensing observed using solutions with different known refractive indexes. From these measurements a sensitivity of 126 nm/RIU was obtained. Moreover, the monitorization of protein absorption onto gold surfaces in a real-time label-free fashion using the nanoholes arrays has demonstrated the biosensing capabilities of the fabricated device.

## References

[1] Nice E.C., Catimel B. (2009). Instrumental biosensors: New perspectives for the analysis of biomolecular interactions. BioEssays.

[2] Roh S., Chung T., Lee B. (2011). Overview of the characteristics of micro- and nano-structured surface plasmon resonance sensors. Sensors.

[3] Ebbesen T.W., Lezec H.J., Ghaemi H.F., Thio T., Wolff P.A. (1998). Extraordinary optical transmission through sub-wavelength hole arrays. Nature.

[4] Thio T., Ghaemi H.F., Lezec H.J., Wolff P.A., Ebbesen T.W. (1999). Surface-plasmon-enhanced transmission through hole arrays in Cr films. J. Opt. Soc. Am. B.

[5] De Leebeeck A., Kumar L.K.S., de Lange V., Sinton D., Gordon R., Brolo A.G. (2007). On-chip surface-based detection with nanohole arrays. Anal. Chem..

[6] Brolo A.G., Gordon R., Leathem B., Kavanagh K.L. (2004). Surface plasmon sensor based on the enhanced light transmission through arrays of nanoholes in gold films. Langmuir.

[7] Lesuffleur A., Im H., Lindquist N.C., Oh S.-H. (2007). Periodic nanohole arrays with shape-enhanced plasmon resonance as real-time biosensors. Appl. Phys. Lett..

[8] Xue J., Zhou W., Dong B., Wang X., Chen Y., Huq E., Zeng W., Qu X., Liu R. (2009). Surface plasmon enhanced transmission through planar gold quasicrystals fabricated by focused ion beam technique. Microelectron. Eng..

[9] Sharpe J.C., Mitchell J.S., Lin L., Sedoglavich N., Blaikie R.J. (2008). Gold nanohole array substrates as immunobiosensors. Anal. Chem..

[10] Kelf T., Sugawara Y., Cole R.M., Baumberg J.J. (2006). Localized and delocalized plasmons in metallic nanovoids. Phys. Rev. B.

[11] Henzie J., Lee J., Lee M.H., Hasan W., Odom T.W. (2009). Nanofabrication of plasmonic structures. Annu. Rev. Phys. Chem..

[12] Chou S.Y., Krauss P.R., Renstrom P.J. (1996). Nanoimprint lithography. J. Vac. Sci. Technol. B.

[13] Semiconductor Industry Association (2010). The International Technology Roadmap for Semiconductors.

[14] Chen J., Shi J., Decanini D., Cambril E., Chen Y., Haghiri-Gosnet A.-M. (2009). Gold nanohole arrays for biochemical sensing fabricated by soft UV nanoimprint lithography. Microelectron. Eng..

[15] Im H., Lee S.H., Wittenberg N.J., Johnson T.W., Lindquist N.C., Nagpal P., Norris D.J., Oh S. (2011). Template-stripped smooth Ag nanohole arrays with silica shells for surface plasmon resonance biosensing. ACS Nano.

[16] Lesuffleur A., Im H., Lindquist N.C., Lim K.S., Oh S. (2008). Laser-illuminated nanohole arrays for multiplex plasmonic microarray sensing. Opt. Express.

[17] Lesuffleur A., Im H., Lindquist N.C., Lim K.S., Oh S. (2008). Plasmonic nanohole arrays for real-time multiplex biosensing. Biosens. Proc. SPIE.

